# Clinical Guidelines and Management of Ankyloglossia with 1-Year Followup: Report of 3 Cases

**DOI:** 10.1155/2013/185803

**Published:** 2013-01-29

**Authors:** Mayur S. Bhattad, M. S. Baliga, Ritika Kriplani

**Affiliations:** Department of Pedodontics and Preventive Dentistry, Sharad Pawar Dental College and Hospital, Sawangi 442001, Wardha, Maharashtra, India

## Abstract

The tongue is an important oral structure that affects speech, position of teeth, periodontal tissue, nutrition, swallowing, nursing, and certain social activities. Ankyloglossia (tongue tie) is a congenital anomaly characterized by an abnormally short, thick lingual frenulum which affects movement of tongue. Though the effect of ankyloglossia in general appears to be a minor condition, but a major difference exists concerning the guidelines for tongue-tie division. There are no accepted practical criteria for the management of such condition, and hence this paper aims at bringing all the compilation in examination, diagnosis, treatment, and management of tongue tie together for better clinical approach.

## 1. Introduction

The tongue is an important organ that affects speech, position of the teeth, periodontal tissue, nutrition, and swallowing [1]. Most of us think of tongue tie as a situation we find ourselves in when we are too excited to speak. Tongue tie is the nonmedical term for a relatively common physical condition that limits the use of the tongue, which is actually called as ankyloglossia [2].

Before birth, a strong cord of tissue guides the development of oral frenulum which is positioned in the centre of the mouth. After birth, this lingual frenulum continues to guide the position of erupting teeth. As the child grows, it recedes and becomes thin. This frenulum is visible when we look at the mirror under the tongue. In some children, the frenulum is especially tight, or it fails to recede and may cause tongue immobility [2]. Hence ankyloglossia is defined as a developmental anomaly of the tongue characterized by an abnormally short, thick lingual frenum resulting in limitation of tongue movement [3], or in simple terms, tongue tie is present when the lingual frenulum is attached close to the tongue tip, resulting in reduced tongue movement.

Various studies using different diagnostic criteria found a prevalence of ankyloglossia between 4 and 10% [4, 5], and the incidence of tongue tie varies from 0.2% to 5% depending on the population examined [3]. It is more common in males, with male to female ratio of 2.5 : 1.0 [5]. Ankyloglossia in infants has an incidence rate from 25% to 60%, and its presence can lead to difficulty in breastfeeding ranging from failure to thrive to even refusing the breast [4, 6–8].

Ankyloglossia can also be a part of certain rare syndromes like Smith-Lemli-Opitz syndrome, orofacial digital syndrome, Beckwith Weidman syndrome, Simpson-Golabi-Behmel syndrome, and X-linked cleft palate with autosomal dominant or recessive trait [5, 9–12].

Ankyloglossia in children poses a diagnostic challenge for dentists. Recent reviews have revealed very minimal information about what constitutes an abnormal lingual attachment and what criteria should be used to justify surgical intervention. Hence the purpose of this report is to describe ankyloglossia, its clinical significance, and what guidelines should be followed before planning surgical intervention.

## 2. Case Reports

**Figure 1:**
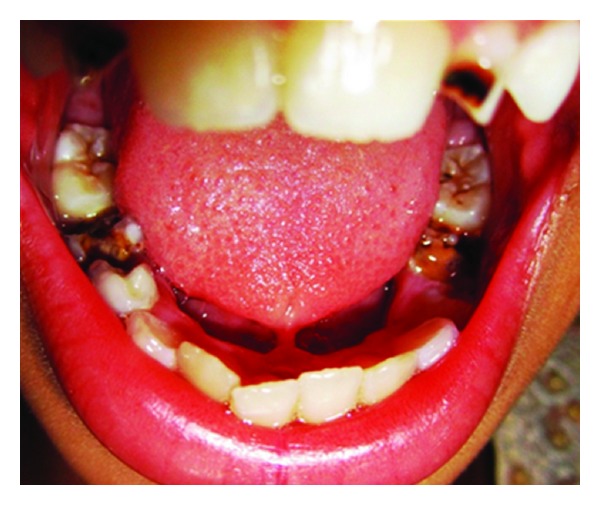
Preoperative photograph (Case 1).

*Case Number *1. A 12-year-old female patient reported to the Department of Pedodontics and Preventive Dentistry with the chief complaint of pain in lower right and left posterior region. Oral examination of the patient revealed not only multiple decayed teeth in lower arch but also an ankyloglossia with thick, short frenulum, restricted tongue protrusion, and lifting of the tip of the tongue (Figure 1).

**Figure 2:**
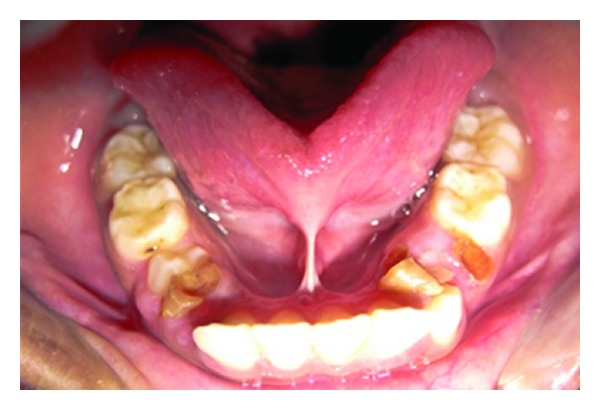
Preoperative photograph (Case 2).

*Case Number *2. An 8-year-old male patient reported to the Department of Pedodontics and Preventive Dentistry with the chief complaint of pain in upper right posterior region. After clinical examination, decayed tooth and ankyloglossia with restricted tongue movements were also observed. A bifid or heart shape of the anterior tip of the tongue was seen upon attempted extension (Figure 2).

**Figure 3:**
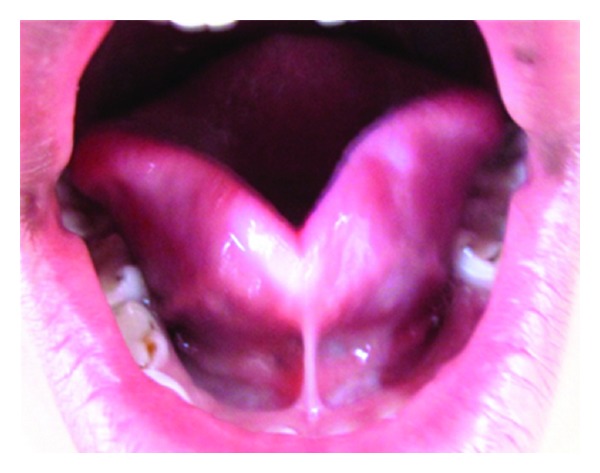
Preoperative photograph (Case 3).

*Case Number *3. An 11-year-old male patient reported to Department of Pedodontics and Preventive Dentistry with the chief complaint of improper speech, and his parents also reported that he was not able to chew solid foods. Clinical examination revealed that patient had ankyloglossia with thick frenum, restricted tongue movements like protrusion, and lifting of the tip of the tongue and a bifid or heart shape of the anterior tip of the tongue, was observed. To assess the extent of limitation of tongue movement, the mouth was carefully inspected under adequate illumination with a tongue depressor (Figure 3).

## 3. Clinical Assessment

All the 3 cases were assessed clinically by Kotlow's criteria (Table 1) in which normal range of motion of the tongue was assessed [1], Hazelbaker's assessment tool (Table 2) to observe the functional movement and appearance of the tongue [13], and speech analysis to identify and rectify defective speech [3, 14].

 Upon diagnosis of an ankyloglossia, the patient's parents were informed about the nature of the lesion, its functional implications, and the variety of surgical approaches. The patient's family and medical history were noncontributory. Patient's height and weight were appropriate for their age. ENT and general physical examination revealed insignificant findings. Hematologic examination of the patients was within normal range. After obtaining informed consent, the following procedures were carried out for correction of lingual frenum.

## 4. Clinical Management

 In the first and second case (cases number 1 and 2), frenum attachment was revised by conventional frenectomy. A topical anesthetic was applied to the underside of the tongue following which block anesthesia was given. After achieving objective symptoms, a suture was passed at the middle of the tongue to control its movements, and two hemostat was used to clamp the frenum: one at the under surface of the tongue and another at the floor of the mouth avoiding salivary gland duct. Incision was placed above and below the hemostats to release the complete frenum. On achieving homeostasis, the area was sutured. The patients were discharged with postoperative instructions.

 In the third case (case number 3), frenum was relieved by using diode lasers. A topical anesthetic was applied to the underside of the tongue. Tongue was raised with the thumb and index finger, and the frenum was revised. After achieving homeostasis, patient was discharged with postoperative instructions.

**Figure 4:**
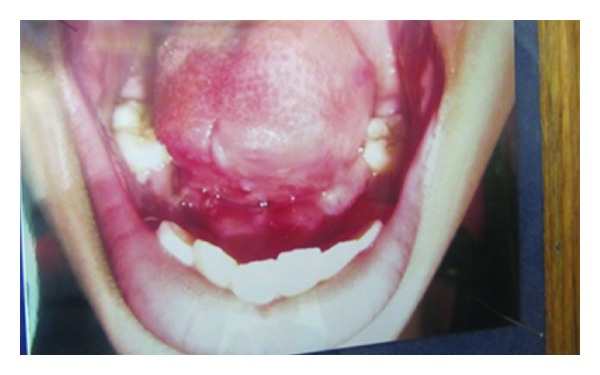
Postoperative photograph after 7 days (Case 1).

**Figure 5:**
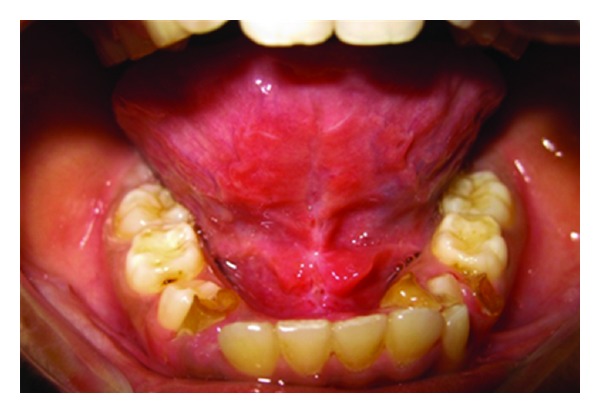
Postoperative photograph after 7 days (Case 2).

**Figure 6:**
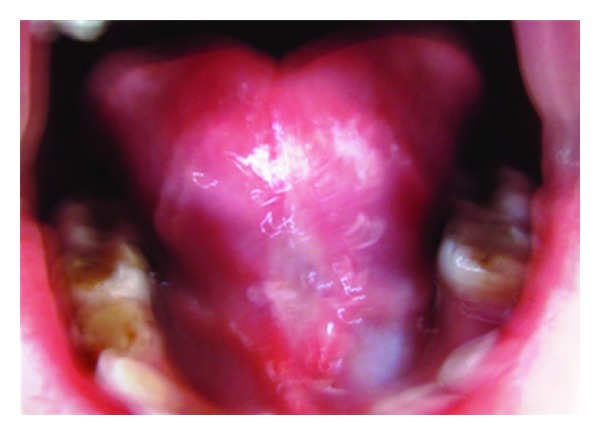
Postoperative photograph after 7 days (Case 3).

 After a week, sutures in all the cases were removed, and case number 3 was referred to speech therapist (Figures 4, 5, and 6). After 1-year followup, all the 3 cases were reassessed again by using the same criterias. 

## 5. Results

**Figure 7:**
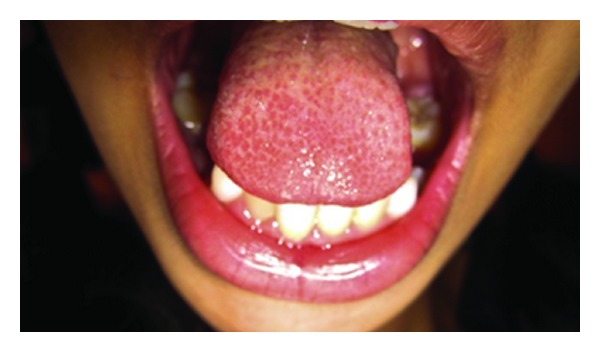
Postoperative photograph after 1 year (Case 1).

**Figure 8:**
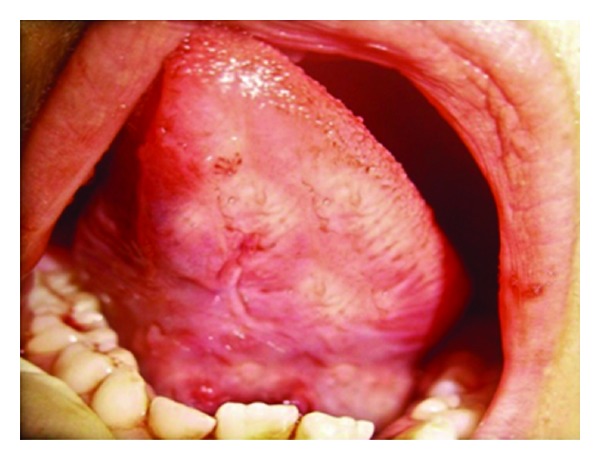
Postoperative photograph after 1 year (Case 2).

**Figure 9:**
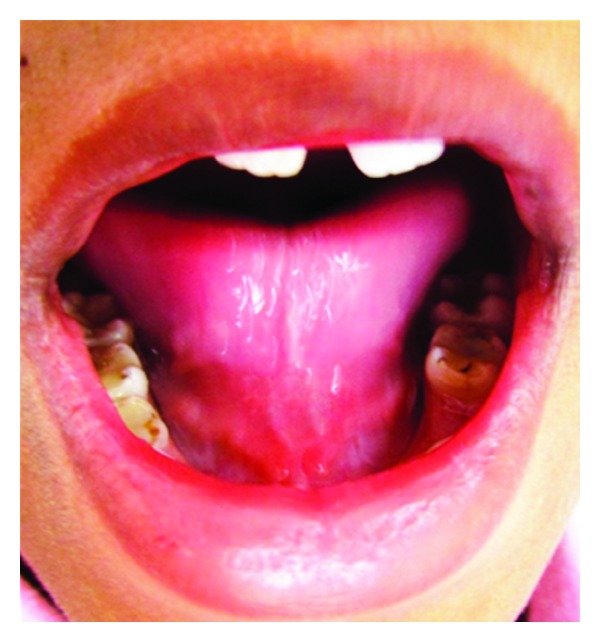
Postoperative photograph after 1 year (Case 3).

 Using the Kotlow's criteria and Hazelbaker's assessment tool, preoperative and postoperative scores were recorded. After 1-year followup, significant improvement in prognosis of symptoms of ankyloglossia was observed (Figures 7, 8, and 9). Free tongue movement increased from 7, 9, and 8 mm to 13, 14, and 15 mm (Table 3), respectively, and functional score of 9, 10, and 10 and appearance score of 6, 6, and 7 were changed to 14, 13, 12, and 9, 9, and 10 (Table 4), respectively. Speech in case number 3 also significantly improved (Table 5). 

## 6. Discussion

Anatomical definition of ankyloglossia consists of descriptions as well as absolute measurements. Descriptions include the attachment of the frenulum to the tongue, the attachment of the frenulum to the inferior alveolar ridge, the elasticity of the lingual frenulum, and the appearance of the tongue when lifted. Absolute measurements include the length of the lingual frenulum when the tongue is lifted as well as the free tongue length [15].

 According to Wallace, functional definition includes it as a condition in which the tip of the tongue cannot be protruded beyond the lower incisor teeth because of a short frenulum. On the other hand, tongue movement is more complex than simple protrusion, and as a result functional assessments criteria have included tongue lateralization, tongue lift, tongue spread, tongue cupping, and tongue snap back [15].

Ankyloglossia can be divided into partial or complete ankyloglossia. The academy of Breastfeeding Medicine Protocol defines partial ankyloglossia as the presence of a sublingual frenulum which changes the appearance and/or function of the infant's tongue because of its decreased length, lack of elasticity or attachment too distal beneath the tongue or too close to or onto the gingival ridge. Complete ankyloglossia is a condition in which there is extensive fusion of the tongue to the floor of the mouth which is extremely rare [16]. 

## 7. Consequences of Not Treating the Tongue Tie

Appearance of the tongue could be abnormal in some individuals. Improper chewing and swallowing of food could increase the gastric distress and bloating, and snoring and bed wetting at sleep are common among tongue tied children. It also affects children who want to participate in routine play which involves tongue movements, gestures, and speech. Dental caries could occur due to food debris not being removed by the tongue's action of sweeping the teeth and spreading of saliva. Malocclusion like open bite due to thrust created by being tongue tied, spreading of lower incisors with periodontitis, and tooth mobility due to long-term tongue thrust are associated problems. It also affects self-esteem because it has been noted clinically that occasionally an older child or adult will be self-conscious or embarrassed about their tongue tie that they may be teased by their classmates for their anomaly. In infant feeding problem may be experienced due to latching on to the nipple which may compress the nipple against the gum resulting in nipple pain in mothers, and due to this the mothers may often try to shift the baby to a bottle [3, 16–18].

## 8. Clinical Guidelines for Management of ****Ankyloglossia

There is a wide difference of opinion regarding its clinical significance and optimal management. In many children, ankyloglossia is asymptomatic, and the condition may resolve spontaneously, or affected children may learn to compensate adequately for their decreased lingual mobility. Some children, however, benefit from surgical intervention of their tongue tie. Parents should be educated about the possible long-term effects of tongue tie, so that they may make an informed choice regarding possible therapy.

 For effective management proper clinical guidelines are mandatory. In ankyloglossia, the most important factor to be considered is the normal range of motion of the tongue which should be determined by using Kotlow's criteria [1] in which classification ranges from class I to class IV. The tip of the tongue should able to protrude outside the mouth without clefting and should be able to sweep the upper and lower lips easily, without straining. When the tongue is retruded, it should not blanch the tissue lingual to the anterior teeth and should not put excessive forces on the mandibular anterior teeth. The lingual frenum should not create a diastema between the mandibular central incisor, and the frenum should not prevent an infant from attaching to the mother's nipple during nursing.

 The functional movement and appearance of the tongue could be determined by using Hazelbakers assessment tool [15]. In this tool, scores are given to each movement of the tongue and appearance of the tongue. If the functional and appearance score is below 11 and 8, then surgical invention should be considered.

 Patients should be asked to pronounce certain words which start from “I,” “th,” “s,” “d,” and “t” to check the accuracy of the word pronunciations. If a defective speech is observed, after postoperative wound healing, referral to a speech therapist is mandatory for speech modification. Postoperative tongue muscle exercises like licking the upper lip, touching hard palate with the tip of tongue, and side-to-side movements should be explained to the patient for enhanced tongue movements. 

## 9. Conclusion

Tongue tie affects a considerable number of infants and children. It is perhaps interesting that such a simple condition can cause such controversy and diversity of opinions. However, it is important that accurate information and guidance is given to parents with regard to the indications and potential benefits of tongue-tie revision, and that appropriate provisions are in place for those infants and children who require revision. These case reports offer guidelines which can be used by general and pediatric dentists for diagnosis and treatment of a tongue restriction resulting from ankyloglossia.

## Figures and Tables

**Table 1:** Kotlow's classification.

Type	Movement of the tongue
Clinically acceptable, normal range of free tongue movement	Greater than 16 mm
Class I: Mild ankyloglossia	12 to 16 mm
Class II: Moderate ankyloglossia	8 to 11 mm
Class III: Severe ankyloglossia	3 to 7 mm
Class IV: Complete ankyloglossia	less than 3 mm

**Table 2:** Hazel baker's Assessment tool for appearance and function of the tongue.

Appearance	Function
Appearance of tongue when lifted	Lateralization

2: Round or square	2: Complete
1: Slight cleft in tip apparent	1: Body or tongue but no tongue tip
0: Heart or V-shaped	0: None

Elasticity of frenulum	Lift of tongue

2: Very elastic	2: Tip to mid-mouth
1: Moderately elastic	1: Only edges to mid-mouth
0: Little or no elasticity	0: Tip stays at lower alveolar ridge or rises to mid-mouth only with jaw closure

Length of lingual frenulum when tongue lifted	Extension of tongue

2: >1 cm	2: Tip over lower lip
1: 1 cm	1: Tip over lower gum only 0: Neither of the above, or anterior or
0: <1 cm	mid-tongue humps

Attachment of lingual frenulum to tongue	Spread of anterior tongue

2: Posterior to tip	2: Complete
1: At tip	1: Moderate of partial
0: Notched tip	0: Little or none

Attachment of lingual frenulum to inferior alveolar ridge	Cupping

2: Attached to floor of mouth or well below ridge	2: Entire edge, firm cup
1: Attached just below ridge	1: Side edges only, moderate cup
0: Attached at ridge	0: Poor or no cup

	Peristalsis

	2: Complete, anterior or posterior
	1: Partial, originating posterior to tip
	0: None or reverse

14 = Perfect score, 11 = Acceptable if appearance item score is 10. Frenectomy is necessary if function score is <11 and appearance score is <8.

**Table 3:** Pre-operative and post-operative assessment of free tongue movement in all the 3 cases by using Kotlow's criterias.

Case number	Pre-operative free tongue movement	Diagnosis	Post-operative, free tongue movement	Diagnosis
1	7 mm	Class III	13 mm	Class I
2	9 mm	Class II	14 mm	Class I
3	8 mm	Class II	15 mm	Class I

**Table 4:** Pre-operative and post-operative assessment of functional and appearance score of all the 3 cases by using Hazel-Baker's assessment tool.

Case number	Pre-operative function score	Pre-operative appearance score	Post-operative function score	Post-operative appearance score
1	9	6	14	9
2	10	6	13	9
3	10	7	12	10

**Table 5:** Pre-operative and post-operative assessment of speech in all the 3 cases.

Case number	Pre-operative associated problem	Post-operative associated problem
1	No speech abnormality	—
2	No speech abnormality	—
3	Defective speech	Improvement of speech

## References

[1] Kotlow LA (1999). Ankyloglossia (tongue-tie): a diagnostic and treatment quandary. *Quintessence International*.

[2] http://entnet.org/HealthInformation/Ankyloglossia.cfm.

[3] Darshan HE, Pavithra PM (2011). Tongue tie: from confusion to clarity-a review. *International Journal of Dental Clinics*.

[4] Segal LM, Stephenson R, Dawes M, Feldman P (2007). Prevalence, diagnosis, and treatment of ankyloglossia: methodologic review. *Canadian Family Physician*.

[5] Saeid M, Mobin Y, Reza R, Ali AP, Mohsen G (2010). Familial ankyloglossia (tongue-tie): a case report. *Acta Medica Iranica*.

[6] Messner AH, Lalakea ML, Macmahon J, Bair E, Janelle A (2000). Ankyloglossia: incidence and associated feeding difficulties. *Archives of Otolaryngology*.

[7] Ballard JL, Auer CE, Khoury JC (2002). Ankyloglossia: assessment, incidence, and effect of frenuloplasty on the breastfeeding dyad. *Pediatrics*.

[8] Tait P (2000). Nipple pain in breastfeeding women: causes, treatment, and prevention strategies. *Journal of Midwifery and Women’s Health*.

[9] Meinecke P, Blunck W, Rodewald A (1987). Smith-Lemli-Opitz syndrome. *American Journal of Medical Genetics*.

[10] Neri G, Gurrieri F, Zanni G, Lin A (1998). Clinical and molecular aspects of the Simpson Golabi Behmel syndrome. *American Journal of Medical Genetics*.

[11] Braybrook C, Doudney K, Marçano ACB (2001). The T-box transcription factor gene TBX22 is mutated in X-linked cleft palate and ankyloglossia. *Nature Genetics*.

[12] Kantaputra PN, Paramee M, Kaewkhampa A (2011). Cleft lip with cleft palate, ankyloglossia, and hypodontia are associated with TBX22 mutations. *Journal of Dental Research*.

[13] Hazelbaker AK (1993). *The assessment tool for lingual frenulum function (ATLFF): use in a lactation consultant private practice [thesis]*.

[14] Ketty N, Sciullo PA (1974). Ankyloglossia with psychological implications. *ASDC Journal of Dentistry for Children*.

[15] Johnson RV (2006). Tongue-tie—exploding the myths. *Infant*.

[16] Ballard J, Chantry C, Howard CR Guidelines for the evaluation and management of neonatal ankyloglossia and its complications in the breastfeeding dyad. http://www.bfmed.org/Media/Files/Protocols/ankyloglossia.pdf.

[17] Tuli A, Singh A (2010). Monopolar diathermy used for correction of ankyloglossia. *Journal of Indian Society of Pedodontics and Preventive Dentistry*.

[18] Kupietzky A, Botzer E (2005). Ankyloglossia in the infant and young child: clinical suggestions for diagnosis and management. *Pediatric Dentistry*.

